# Targeted dual inhibition of c‐Met/VEGFR2 signalling by foretinib improves antitumour effects of nanoparticle paclitaxel in gastric cancer models

**DOI:** 10.1111/jcmm.16362

**Published:** 2021-05-03

**Authors:** Meghan Grojean, Margaret A. Schwarz, Johann R. Schwarz, Sazzad Hassan, von Holzen U, Changhua Zhang, Roderich E. Schwarz, Niranjan Awasthi

**Affiliations:** ^1^ Department of Surgery Indiana University School of Medicine South Bend IN USA; ^2^ Department of Pediatrics Indiana University School of Medicine South Bend IN USA; ^3^ Harper Cancer Research Institute University of Notre Dame South Bend IN USA; ^4^ Goshen Center for Cancer Care Goshen IN USA; ^5^ University of Basel Basel Switzerland; ^6^ Department of Gastrointestinal Surgery The Seventh Affiliated Hospital of Sun Yat‐sen University Shenzhen China; ^7^ Roswell Park Comprehensive Cancer Center Buffalo NY USA; ^8^ University of Buffalo Buffalo NY USA

**Keywords:** c‐Met, combination therapy, foretinib, gastric cancer, *nab*‐paclitaxel

## Abstract

Elevated expression of multiple growth factors and receptors including c‐Met and VEGFR has been reported in gastric adenocarcinoma (GAC) and thus provides a potentially useful therapeutic target. The therapeutic efficacy of foretinib, a c‐Met/VEGFR2 inhibitor, was determined in combination with nanoparticle paclitaxel (NPT) in GAC. Animal studies were conducted in NOD/SCID mice in subcutaneous and peritoneal dissemination xenografts. The mechanism of action was assessed by Immunohistochemical and Immunoblot analyses. In c‐Met overexpressing MKN‐45 cell‐derived xenografts, NPT and foretinib demonstrated inhibition in tumour growth, while NPT plus foretinib showed additive effects. In c‐Met low‐expressing SNU‐1 or patient‐derived xenografts, the foretinib effect was smaller, while NPT had a similar effect compared with MKN‐45, as NPT plus foretinib still exhibited an additive response. Median mice survival was markedly improved by NPT (83%), foretinib (100%) and NPT plus foretinib (230%) in peritoneal dissemination xenografts. Subcutaneous tumour analyses exhibited that foretinib increased cancer cell death and decreased cancer cell proliferation and tumour vasculature. NPT and foretinib suppressed the proliferation of GAC cells in vitro and had additive effects in combination. Further, foretinib caused a dramatic decrease in phosphorylated forms of c‐Met, ERK, AKT and p38. Foretinib led to a decrease in Bcl‐2, and an increase in p27, Bax, Bim, cleaved PARP‐1 and cleaved caspase‐3. Thus, these findings highlight the antitumour impact of simultaneous suppression of c‐Met and VEGFR2 signalling in GAC and its potential to enhance nanoparticle paclitaxel response. This therapeutic approach might lead to a clinically beneficial combination to increase GAC patients’ survival.

## 1 INTRODUCTION

Gastric adenocarcinoma (GAC) is one of the most frequent and deadly malignancies in the world, even after a steady decrease in the number of cases. Clinical outcome of GAC patients is poor and it remains the 3rd most frequent cause of cancer mortality worldwide.^1^ The treatment of GAC primarily depends on the tumour stage and surgical removal offers the best option of treatment in early tumour stages. Most GAC cases are diagnosed in advanced stages where systemic chemotherapy is a preferred treatment. Different chemotherapy regimens have been used for patients with advanced metastatic or recurrent GAC leading to a median survival time of less than a year.^2^, ^3^, ^4^ Recently, a triple chemotherapy regimen, docetaxel plus oxaliplatin and 5‐FU (FLOT), became a standard 1st‐line option for gastric cancer patients based on its superior clinical response.^5^ Taxanes and irinotecan are generally recommended as a 2nd‐line therapy for GAC. Unfortunately, these intensive therapies demonstrate a meaningful clinical improvement in fewer than half of the patients and a significant proportion of patients remain unbenefited.^6^ Further, many patients who initially respond to these systemic therapies, develop chemoresistance and relapse.^7^ Thus, there is a compelling requirement for novel therapeutic options that can improve the outcomes of GAC.

Nanoparticle albumin‐bound paclitaxel (*nab*‐paclitaxel, NPT) has markedly improved safety profile and antitumour activity based on its intrinsically enhanced permeability and retention, accumulation and penetration in the tumour. The FDA has approved *nab*‐paclitaxel for the treatment of breast cancer, non‐small cell lung cancer (NSCLC) and pancreatic cancer.^8^ Earlier studies from our laboratory showed greater antitumour activity of *nab*‐paclitaxel compared with other routinely used chemotherapies in GAC animal models.^9^ In this study, a 3rd‐generation platinum chemotherapy drug oxaliplatin (Oxa) was used as a reference control because platinum chemotherapy drugs have been commonly used for advanced GAC treatment.^10^, ^11^

As the therapeutic efficacy of cytotoxic agents remains limited, molecular analysis of GAC may provide novel therapeutic targets that could improve outcomes. Over the last decade, targeted therapies have indeed made some progress in GAC treatment. In 2010, an epidermal growth factor receptor 2 (HER2) antibody trastuzumab was approved for HER2‐positive metastatic GAC.^12^ In 2014, the vascular endothelial growth factor receptor 2 (VEGFR2) antibody ramucirumab was approved as a single‐agent or combined with paclitaxel for advanced GAC.^13^, ^14^ Ramucirumab's approval for GAC further established the therapeutic importance of VEGF‐induced angiogenesis in this disease. A 2013 study suggested that therapies targeting receptor tyrosine kinase (RTK)/RAS pathways could have therapeutic potential in approximately 37% of GAC cases.^15^ These facts corroborate the therapeutic potential of targeted agents in GAC.

Gastric cancer epithelial and stromal cells in the tumour microenvironment release an extensive variety of growth factors and their receptors that confer several oncogenic changes including angiogenesis, invasion and metastasis.^16^ Aberrantly activated or overexpressed hepatocyte growth factor (HGF) and its receptor (c‐Met) pathway have been manifested in many cancers including GAC.^17^, ^18^, ^19^ Previously published reports demonstrated the involvement of abnormal c‐Met signalling in several oncogenic pathways such as proliferation, invasion and angiogenesis; and it has been indicated as an independent prognostic factor for the worst outcome in many cancers.^17^, ^20^, ^21^, ^22^ These reports provide a strong rationale for targeting c‐Met in GAC treatment. Various inhibitors of c‐Met signalling have been investigated in GAC treatment. Tivantinib as monotherapy demonstrated modest clinical efficacy in Asian patients with metastatic GAC; however, there were some serious adverse events.^23^ Another c‐Met inhibitor SAR125844 also demonstrated limited antitumour activity in Met‐positive GAC patients in a phase I study.^24^ In preclinical models, several c‐Met inhibitors including SU11274, KRC‐408, Simm530 and T‐1840383 displayed an antineoplastic effect in gastric cancer.^25^, ^26^, ^27^, ^28^ Similar to c‐Met, VEGFR2 is a cardinal angiogenic factor associated with GAC progression. Previous reports demonstrated the correlation between elevated VEGF and GAC progression, aggressiveness, early recurrence and dismal survival.^29^, ^30^ Pennacchietti et. al. reported the crosstalk between c‐Met and VEGFR2 pathways as both are induced and regulated by tumour hypoxia.^31^ In an NSCLC preclinical study, the HGF/c‐Met pathway was shown to be involved in VEGF inhibitor resistance.^32^ These findings indicate the importance of concurrent suppression of c‐Met and VEGFR2 pathways to achieve a meaningful antitumour response in GAC.

Foretinib (FTB, Figure S1) is a multikinase inhibitor that blocks c‐Met (IC50: 0.4 nM) and VEGFR2 (IC50: 0.9 nM) pathways with high affinity and other pathways such as Axl, PDGFR‐β, c‐Kit, Flt‐3 and Tie2 with lower affinity.^33^ In preclinical models, foretinib exhibited significant antitumour potential in many cancers.^34^, ^35^, ^36^, ^37^ Foretinib has also been evaluated for its antitumour activity in several clinical trials (Clinicaltrails.gov).

In this study, we sought to investigate the antitumour efficacy of dual targeting of c‐Met and VEGFR2 pathways, and its potency to improve *nab*‐paclitaxel response, in diverse gastric cancer preclinical models.

## 2 MATERIALS AND METHODS

### 2.1 Reagents

*Nab*‐paclitaxel (Celgene Corporation, Summit, NJ) was procured from the Goshen Center for Cancer Care Pharmacy (Goshen, IN). Foretinib was purchased from LC Laboratories (Woburn, MA). The cell proliferation reagent WST‐1 was purchased from Sigma‐Aldrich.

### 2.2 Cell culture

The human GAC cell lines SNU‐1 and KATO‐III were purchased from the American Type Culture Collection (ATCC, Rockville, MD). MKN‐45 GAC cell line was obtained from Creative Bioarray (Shirley, NY). The characteristics of these GAC cell lines are presented in Table S1 indicating that MKN‐45 and KATO‐III cells express c‐Met oncogene, while SNU‐1 cells are c‐Met wild‐type. All GAC cells were grown in RPMI 1640 medium (Sigma) containing 10% or 20% FBS and maintained at 37°C in a humidified incubator with 5% CO_2_ and 95% air.

### 2.3 Cell viability assay

In vitro cell viability assays were executed by adding the colorimetric WST‐1 reagent. Four to five thousand cells were plated in each well of a 96‐well plate in the regular growth medium. The medium was replaced after 16 hours with 2% FBS containing medium and the cells were treated with *nab*‐paclitaxel, oxaliplatin or foretinib. WST‐1 reagent (10 μl) was added to each well after 72‐hour incubation followed by an additional 2 hours incubation. In a microplate reader, the absorbance of each sample was measured at 450 nm.

### 2.4 Western blot analysis

Cell monolayers were treated with *nab*‐paclitaxel and foretinib, incubated for 16 hours and whole cell lysates were prepared. Tumour lysates were prepared as previously described.^38^ Briefly, tumour tissues from subcutaneous xenografts were snap‐frozen in liquid nitrogen and stored at −80°C. These tissues were suspended in lysis buffer containing protease and phosphatase inhibitors and homogenized in a Bullet Blender Homogenizer (Next Generation, Averill Park, NY), and extracts were sonicated. Proteins were separated on 10% polyacrylamide gels by electrophoresis and immobilized by transfer to a PVDF membrane (Bio‐Rad, Hercules, CA). Membranes were incubated overnight at 4°C with the following antibodies: AKT, Phospho‐AKT (Ser473), ERK1/2, phospho‐ERK1/2 (Thr202/Tyr204), c‐Met, Phospho‐c‐Met (Tyr1234/1235), cleaved PARP, cleaved caspase‐3, Bcl‐xl, Bim, GAPDH (Cell Signaling Technology, Beverly, MA); Bcl‐2 and Bax (Santa Cruz Biotechnologies, Dallas, TX). This is followed by incubation with the corresponding HRP‐conjugated secondary antibodies (Pierce Biotechnologies, Santa Cruz, CA) for 1 to 2 hours. Protein bands were visualized using the enhanced chemiluminescence reagent in an Image360 system and densitometry analysis was performed.

### 2.5 In vivo studies

Animal experiments were performed following the Institutional Animal Care and Use Committee (IACUC) guidelines of the Indiana University School of Medicine (South Bend, IN). Mice were housed within the specific pathogen‐free facility and were given ad libitum access to food and water. Female NOD/SCID mice aged 4‐6 weeks were purchased from Charles River Laboratories (Wilmington, MA).

Cell‐derived subcutaneous xenograft (CDX) model: Human GAC cells MKN‐45 (5 x 10^6^) or SNU‐1 (10 x 10^6^) were implanted subcutaneously into the right flank region of NOD/SCID mice. After 10 days, mice were randomly divided into six groups (n = 5) and injected intraperitoneally with PBS (control), *nab*‐paclitaxel (10 mg/kg, twice a week), oxaliplatin (5 mg/kg, twice a week) or foretinib (30 mg/kg, 3 times a week) for 2 weeks.

Patient‐derived subcutaneous xenograft (PDX) model: A poorly differentiated diffuse‐type gastric cancer patient‐derived tumour was obtained from Celprogen (Torrance, CA). Tumour tissue was sectioned into 1 mm^3^ pieces and engrafted subcutaneously into the right flanks of NOD/SCID mice using a Trocar needle. After 3 weeks, the PDX‐bearing mice were randomly divided into four groups (n = 5) and intraperitoneally injected with PBS (control), *nab*‐paclitaxel (10 mg/kg, twice a week) or foretinib (30 mg/kg, 3 times a week) for 2 weeks.

In both CDX and PDX studies, the tumour size was measured twice weekly, and tumour volume (V) was calculated using the formula V = ½ (Length x Width^2^). After 2 weeks of therapy, mice were euthanized; tumours dissected and processed for immunohistochemical and Immunoblot analysis.

### 2.6 Immunohistochemical analysis

Tumour sections obtained from subcutaneous xenografts were fixed in 4% paraformaldehyde, embedded in paraffin and sectioned. Tumour sections (5 μm) were deparaffinized and rehydrated followed by heat‐mediated antigen retrieval in citrate buffer. The sections were then blocked by CAS buffer for 20 minutes. Tumour cell proliferation was measured by overnight incubation with anti‐Ki67 antibody (Abcam, Cambridge, MA) at 4°C and 40 minutes incubation at room temperature with Cy3 secondary antibody. Slides were mounted using a fluorescence mounting solution and imaged under a fluorescence microscope. Tumour cell proliferation was evaluated by counting Ki67‐positive cells from five different high‐power fields (HPF) in a blinded manner. Detection of apoptotic cells in tumour sections was performed by the TUNEL method using ‘Apoptag Apoptosis Detection Kit’ according to the manufacturer's (Millipore) protocol. Microvessel density was determined by staining tumour sections with endomucin antibody (Millipore; MAB2624) at 4°C followed by Cy3 secondary antibody incubation for 40 minutes at room temperature. Slides were mounted using a fluorescence mounting solution and imaged under a fluorescence microscope. Microvessel density was examined by counting endomucin positive vessels in five representative HPF in a blinded manner. Olympus microscope IX81 was used to perform fluorescence microscopy and images were captured with a Hamamatsu Orca digital camera (Hamamatsu Corporation, Bridgewater, NJ) with a DSU spinning confocal unit using cellSens Dimension software (Olympus, Center Valley, PA).

### 2.7 Animal survival analysis

Animal survival studies were performed in a peritoneal dissemination xenograft model using 4‐6‐week‐old female NOD/SCID mice as previously described.^39^ Gastric cancer cells MKN‐45 (10x10^6^) were injected into the peritoneal cavity of mice. Ten days after tumour cell injection, mice were randomized (6‐8 mice per group) to receive PBS (control), *nab*‐paclitaxel, oxaliplatin and foretinib for 2 weeks as described in the subcutaneous experiment. Moribund mice were euthanized when they meet predefined criteria, including rapid weight loss of 15‐20 percent, tumours exceeding 2 cm in any direction, lethargy, inability to remain upright or lack of strength. The survival of the mice was evaluated from the first day of treatment until death.

### 2.8 Statistical analysis

The two‐tailed Student's t test (GraphPad Prism 6.0 Software, San Diego, CA) was used to analyse statistical significance for the individual group comparison. For in vivo tumour growth studies, statistical analysis was executed by one‐way ANOVA for multiple group comparisons and Student's t test for the individual group comparisons. Nonparametric testing with log‐rank group comparisons (GraphPad Prism 6.0) was applied for survival study statistics. In vitro cell proliferation data are expressed as the mean ± standard deviation. Values of *P* < 0.05 were considered to represent statistically significant group differences.

## 3 RESULTS

### 3.1 Foretinib inhibits tumour growth and augments *nab*‐paclitaxel response

**FIGURE 1:**
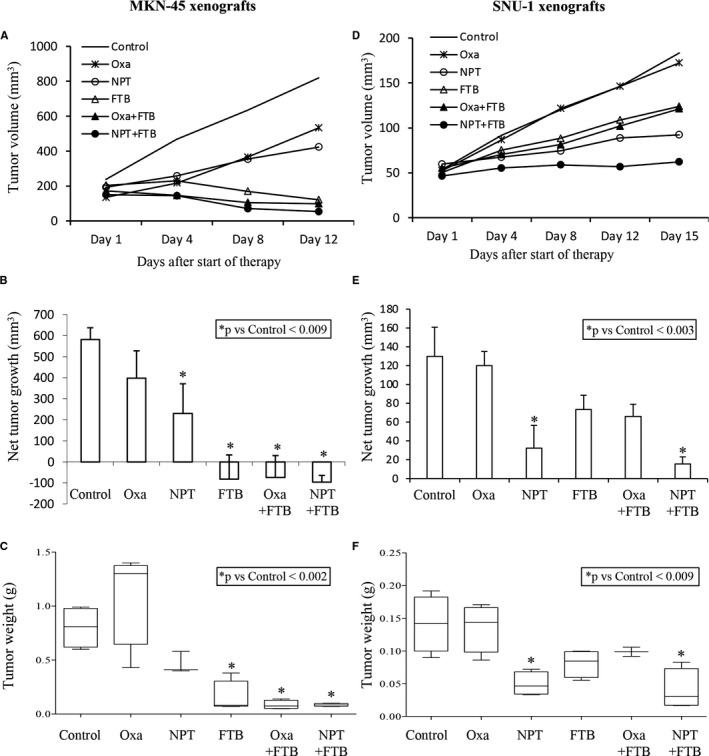
Foretinib inhibits GAC cell‐derived xenograft tumour growth and augments *nab*‐paclitaxel response. Subcutaneous xenograft tumour growth using MKN‐45 cells A, B and C, or SNU‐1 cells D, E and F. Ten days after tumour cell injection mice were treated with foretinib, oxaliplatin and *nab*‐paclitaxel for 2 wk. A and D, Measurements of xenograft tumours were performed twice per week and data are plotted. B and E, Net impact on tumour size was calculated by subtracting tumour volume on the first therapy day from that on the last day. C and F, Mean tumour weights at the end of the experiment presented as box‐and‐whisker plots. Data are representative of the mean values ± standard deviation. Statistical analysis was carried out by Student's t test for the individual group comparison and one‐way ANOVA for multiple group comparisons

In cell‐derived subcutaneous xenografts using MKN‐45 cells that overexpress the c‐Met oncogene, compared with control (PBS treated), tumour size reduction by oxaliplatin was small (32%), while *nab*‐paclitaxel caused a higher tumour size reduction (60.5%). Foretinib monotherapy exhibited a marked tumour regression response and tumour size decreased to 59.4% of its original value. Foretinib combination with *nab*‐paclitaxel showed an additive effect on tumour regression as tumour size decreased to 35.8% of its original value (Figure 1A, 1B). Reduction in tumour weight by mono‐ and combination therapies correlated with the tumour growth inhibition findings and validated the therapeutic advantages of the two drugs (Figure 1C).

In SNU‐1 cell line derived subcutaneous xenografts that do not overexpress c‐Met, there was no notable effect on tumour size by oxaliplatin, while single‐agent therapy with *nab*‐paclitaxel and foretinib decreased tumour size by 76% and 46%, respectively. While tumour size reduction in this case after NPT + FTB was also higher than after monotherapies, foretinib alone or in combination caused less tumour size reduction compared with MKN‐45 xenografts (Figure 1D, 1E). Again, tumour weight data was nicely correlated with the mean tumour volume data to demonstrate antitumour benefits of the two drugs (Figure 1F). In both the subcutaneous tumour experiments, the mice body weight did not change considerably in all groups throughout the therapy period (Figure S2).

### 3.2 Foretinib improves animal survival and augments survival benefits of *nab*‐paclitaxel

**FIGURE 2:**
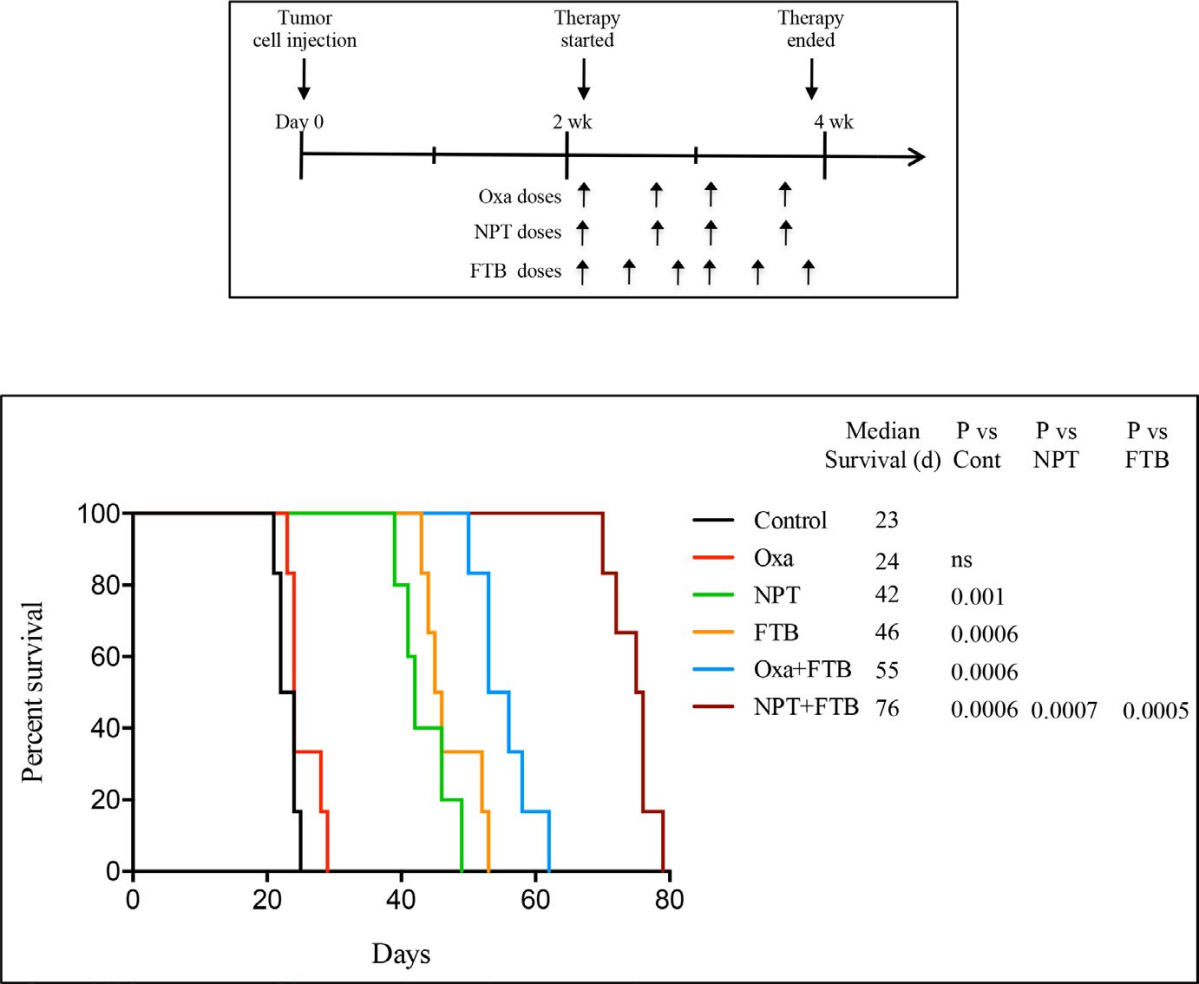
Foretinib enhances animal survival and improves *nab*‐paclitaxel benefits. A, Experimental procedure of survival study in the MKN‐45 cell line‐derived peritoneal dissemination model. Ten days after tumour cell injection, mice were treated with foretinib, oxaliplatin and *nab*‐paclitaxel for the next 2 wk. B, Kaplan‐Meier survival curve representing the mice survival time from the beginning of treatment. Log‐rank testing was used to calculate statistical group differences in survival time

In peritoneal dissemination xenografts using MKN‐45 cells, control mice (PBS‐treated) had a median survival time of 23 days. Control moribund mice had tumours in the stomach, gastroesophageal junction and post‐pyloric duodenum, and metastases were detected in the liver, lungs and gallbladder. Compared with control mice, oxaliplatin did not cause any improvement in median survival (24 days), but mice survival was markedly improved by *nab*‐paclitaxel (42 days, an 83% extension) and foretinib monotherapy (46 days, a 100% extension). Animal survival was further improved by the addition of foretinib to oxaliplatin and *nab*‐paclitaxel: Oxa + FTB (55 days, a 139% extension) and NPT + FTB (76 days, a 230% extension) (Figure 2).

### 3.3 Foretinib inhibits tumour growth in PDX model and augments *nab*‐paclitaxel effects

**FIGURE 3:**
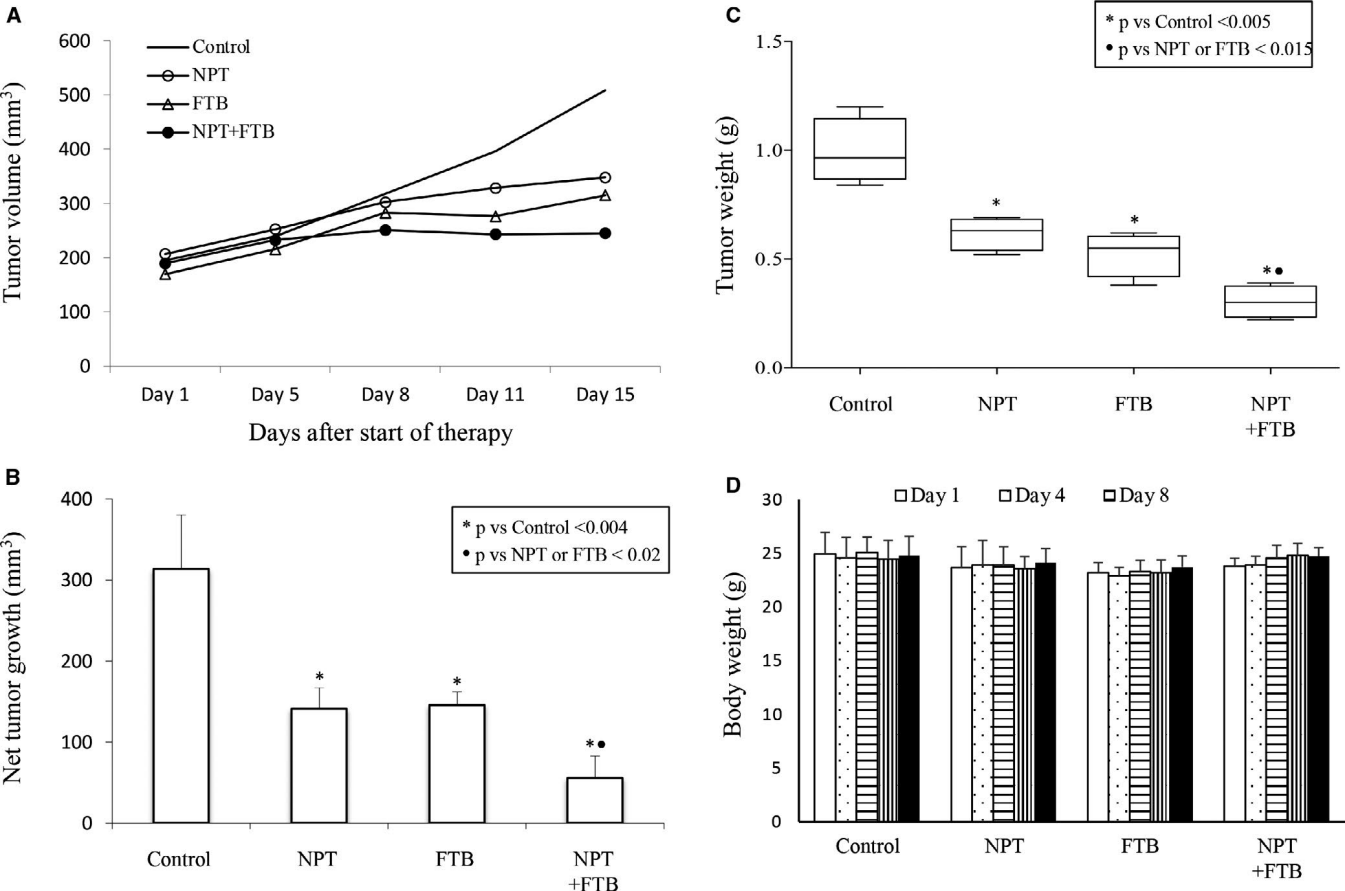
Foretinib reduces tumour growth and augments *nab*‐paclitaxel response in GAC patient‐derived xenografts. NOD/SCID mice carrying GAC patient‐derived tumours were treated with foretinib and *nab*‐paclitaxel for 2 wk. A, tumour size was quantified twice a week and plotted. B, Net impact on tumour growth was calculated by subtracting tumour volume on the first therapy day from that on the last day and data presented as a bar graph. C, The mean tumour weight at the end of the treatment period plotted as a bar graph. D, Mouse weight during a 2‐week therapy period

In gastric cancer patient‐derived xenografts, foretinib and *nab*‐paclitaxel delayed tumour growth and their combination had an additive effect (Figure 3A). The net enhancement in tumour volume in PBS‐treated control, NPT, FTB and NPT + FTB was 314 mm^3^, 141 mm^3^, 145 mm^3^ and 56 mm^3^, respectively (Figure 3B). Excised tumour weights after 15 days of treatment were consistent with tumour volume and compared with control, reduction in tumour weights were 37.7% in NPT, 47.5% in FTB and 70% in NPT + FTB (Figure 3C). Consistent with cell‐derived xenograft experiments, there was no notable treatment‐related change in mice body weight (Figure 3D).

### 3.4 Foretinib reduces cancer cell proliferation and cell death

**FIGURE 4:**
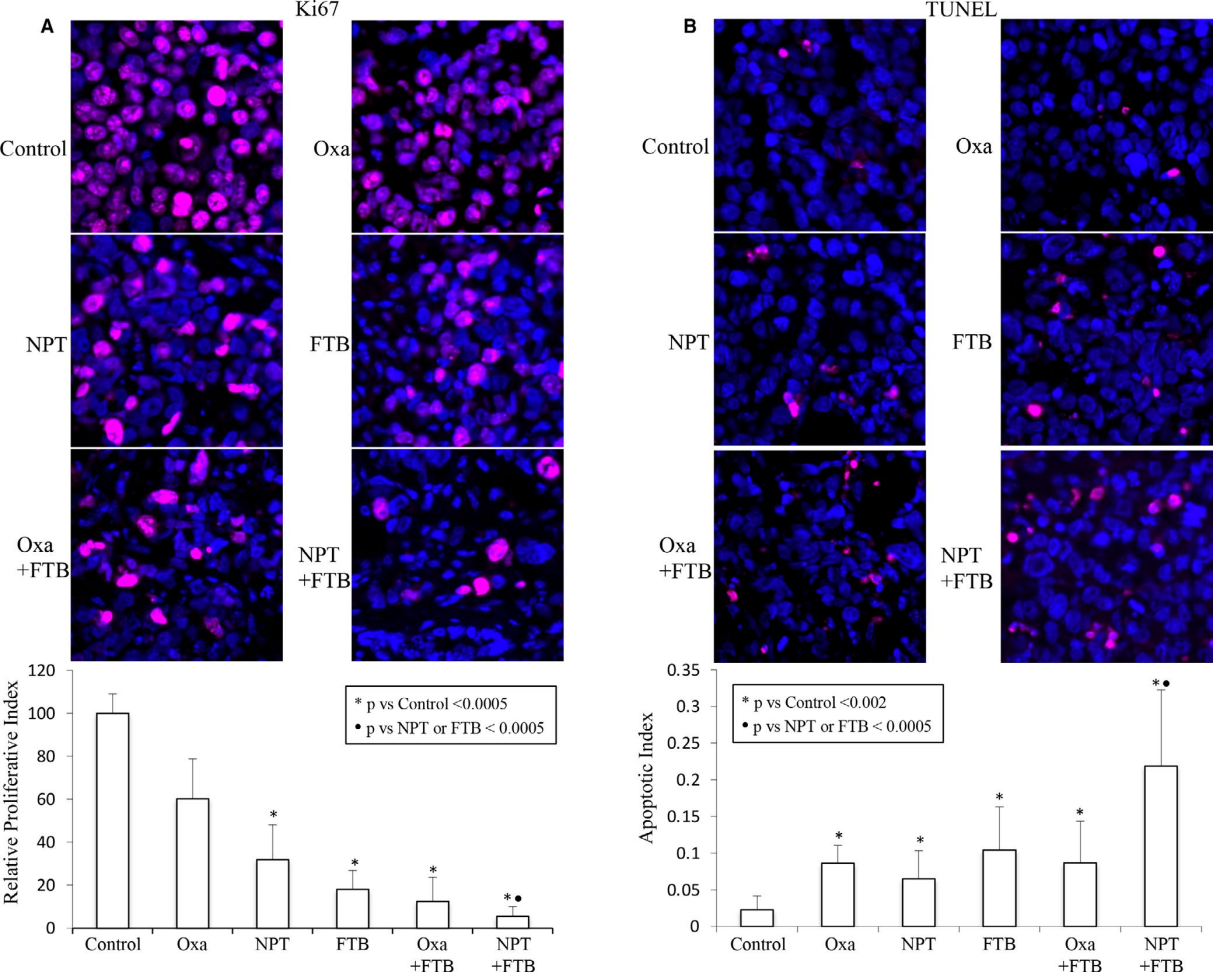
Foretinib and *nab*‐paclitaxel: impact on cancer cell proliferation and cell death. Tumour sections obtained from MKN‐45 cell line‐derived xenografts after a 2‐week therapy with foretinib, oxaliplatin and *nab*‐paclitaxel, were used for the IHC analysis. A, Cancer cell proliferation was determined by immunostaining with Ki67 antibody. Ki67‐stained tumour nuclei were quantified in five different high‐power fields (HPF). B, Cancer cell death was evaluated by staining tumour sections with the TUNEL procedure. The number of apoptotic cells was quantified in at least five HPF. The data are presented as the mean ± standard deviation

Ki67 staining of subcutaneous tumours (MKN‐45 cell‐derived) exhibited that foretinib had the greatest efficacy in attenuating the proliferation of cancer cells (by 82%). *Nab*‐paclitaxel and oxaliplatin decreased proliferation of cancer cells by 68.2% and 39.7%, respectively. A combination of *nab*‐paclitaxel with foretinib (94.5% reduction) was more efficient than monotherapies (Figure 4A).

Cancer cell apoptosis analysis in subcutaneous tumours (MKN‐45 cell‐derived) indicated that in comparison with PBS‐treated control (apoptosis index: 0.02), foretinib (0.104) yielded higher activity than oxaliplatin (0.086) or *nab*‐paclitaxel (0.0.065) monotherapy. The combination of foretinib with oxaliplatin (0.087) was not different than monotherapies, but foretinib plus *nab*‐paclitaxel therapy showed the greatest increase in apoptosis (apoptotic index: 0.219) (Figure 4B).

### 3.5 Foretinib alleviates tumour vasculature and alters expression of marker proteins

**FIGURE 5:**
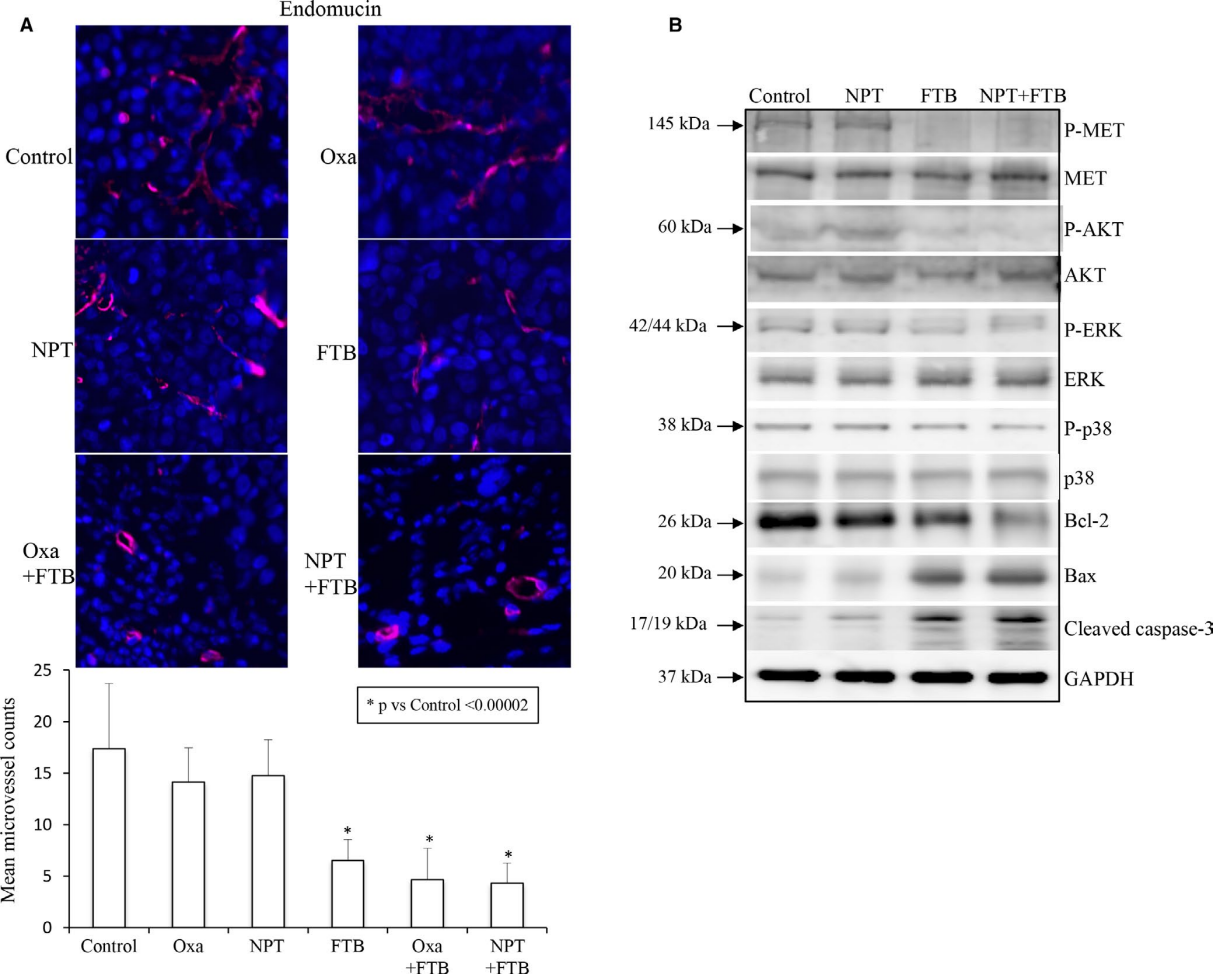
Foretinib and *nab*‐paclitaxel: impact on tumour vasculature and expression of marker proteins. A, Intratumoral microvessel density was determined using MKN‐45 subcutaneous tumours. Tumour sections were incubated with the anti‐endomucin primary antibody and examined by fluorescent microscopy. Endomucin stained blood vessels were counted in at least five HPF and the data are represented as the mean ± standard deviation. B, Tumour lysates were prepared from MKN‐45 subcutaneous xenograft tumours. Tumour lysates of at least 5 mice were pooled in each group and analysed by immunoblotting

Endomucin staining of microvessel in subcutaneous tumours (MKN‐45 cell‐derived) to examine tumour vasculature displayed that oxaliplatin and *nab*‐paclitaxel had no significant effect, in contrast to foretinib that led to a 63% reduction. Furthermore, alleviation in microvessel density in foretinib plus *nab*‐paclitaxel or foretinib plus oxaliplatin therapy was statistically not different from single‐agent foretinib therapy (Figure 5A).

Immunoblot analysis of MKN‐45 subcutaneous tumour lysates demonstrated that foretinib treatment led to a dramatic reduction in phosphorylated c‐Met protein levels and the PI3K/MAPK pathway proteins phospho‐AKT, phospho‐ERK and phospho‐p38. Among Bcl‐2 family proteins, foretinib decreased the levels of anti‐apoptotic protein Bcl‐2 and increased the levels of pro‐apoptotic protein Bax. Foretinib also led to an increase in apoptosis‐markers cleaved PARP‐1 and cleaved caspase‐3. (Figure 5B).

### 3.6 Foretinib inhibits in vitro GAC cell viability

**FIGURE 6:**
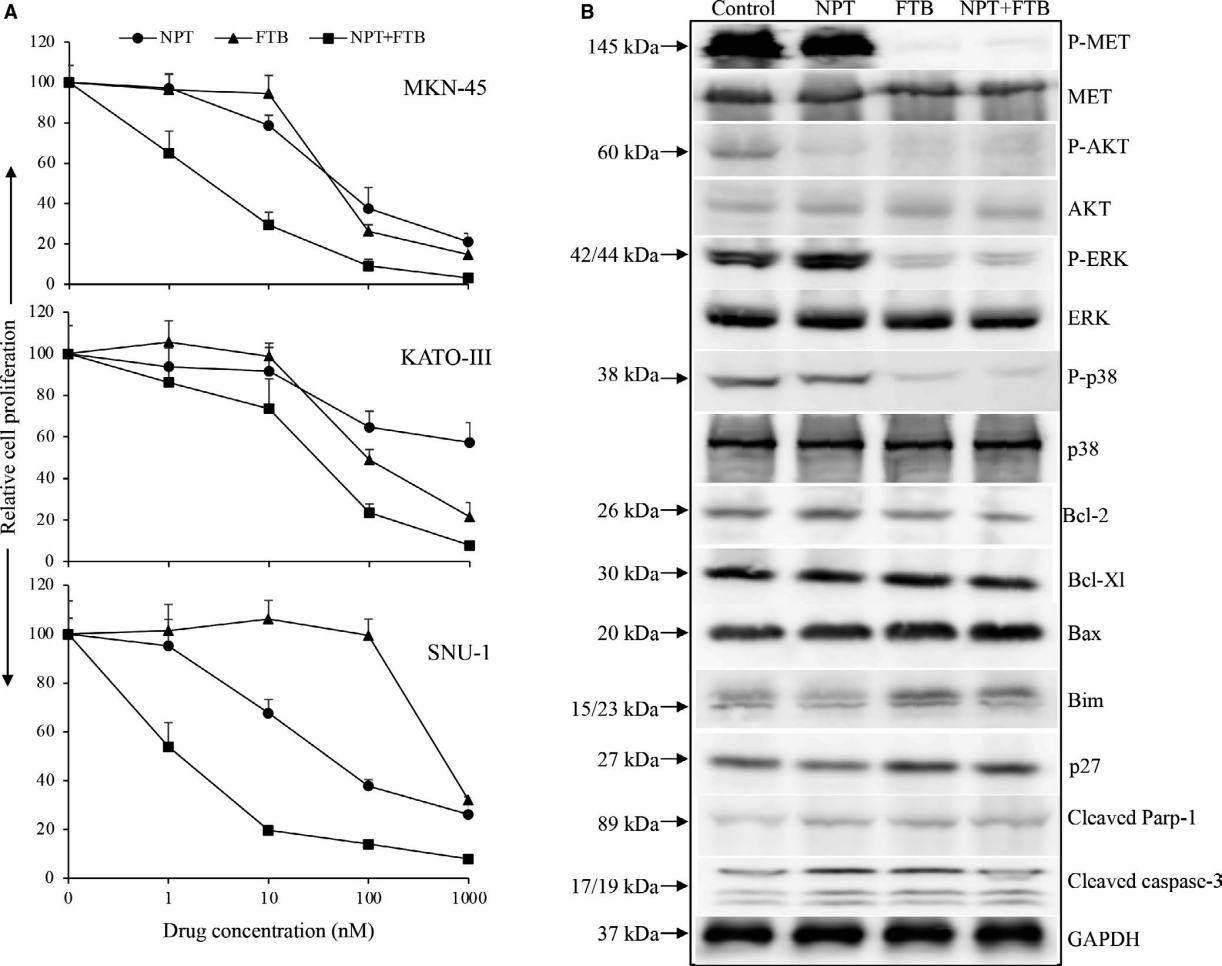
Foretinib and *nab*‐paclitaxel: impact on in vitro proliferation of GAC cells. A, GAC cells (MKN‐45, KATO‐III and SNU‐1) were plated on 96‐well plates and treated with 1 nM, 10 nM, 100 nM and 1000 nM concentrations of foretinib and *nab*‐paclitaxel for 72 h. WST‐1 reagent (10 μl) was added after treatment incubation followed by additional incubation for 2 h. Cell viability was quantitated by measuring the absorbance at 450 nm on a microplate reader. The data are representative of the mean ± standard deviation of triplicate determinations. B, The sub‐confluent MKN‐45 cells were treated with foretinib and *nab*‐paclitaxel for 16 h. Whole cell extracts were prepared, and protein expression levels were analysed by Western blot. The images shown are representative Western blot data of at least two independent experiments with identical results

Foretinib caused an inhibitory effect on the viability of GAC cells in a dose‐proportional manner. In the c‐Met overexpressing GAC cell lines MKN‐45 and KATO‐III, reduction in cell viability at 10 nM, 100 nM and 1 μM concentrations of foretinib were 5.4%, 73.7% and 85.3% for MKN‐45, and 1.2%, 51% and 78.5% for KATO‐III. In the c‐Met low‐expressing GAC cell line SNU‐1, there was no growth inhibitory effect of foretinib at 1 nM, 10 nM or 100 nM, but 68.1% inhibition was observed at a 1 μM concentration (Figure 6A). Single‐agent *nab*‐paclitaxel also led to inhibition in cell viability of all three GAC cell lines tested in a dose‐proportional manner (Figure 6A). Foretinib and *nab*‐paclitaxel combination treatment demonstrated additive inhibitory effects in medium and higher dose groups in this setting, too (Figure 6A).

### 3.7 Foretinib modifies the expression of marker proteins in vitro

Immunoblot analysis of MKN‐45 cell lysates demonstrated that foretinib dramatically alleviated the levels of phospho‐c‐Met protein and the PI3K/MAPK pathway proteins phospho‐AKT, phospho‐ERK and phospho‐p38. Among Bcl‐2 family proteins, foretinib reduced anti‐apoptotic Bcl‐2 protein levels, induced pro‐apoptotic proteins Bax and Bim; and caused no apparent change in Bcl‐xl protein levels. Foretinib also led to an increase in cell cycle inhibitor protein p27 and apoptosis‐marker cleaved PARP‐1 and cleaved caspase‐3 proteins (Figure 6B).

## 4 DISCUSSION

Similar to HER2 and VEGFR2, c‐Met is a RTK that is critically involved in GAC progression, invasion, angiogenesis and metastasis.^40^ In GAC, c‐Met overexpression or gene amplification has been reported in a high percentage of patients, which is associated with aggressive cancer phenotype and worse survival outcome.^41^, ^42^, ^43^ The c‐Met signalling has also been implicated in promoting resistance against anti‐HER2 and anti‐VEGF therapies.^32^, ^44^ Given these insights, it seems logical to explore simultaneous targeting of c‐Met and VEGFR2 pathways in combination with effective chemotherapy with the goal to achieve a more meaningful antitumour response in GAC.

GAC metastasis is mainly induced by peritoneal dissemination and is considered a dominant driver of dismal outcomes.^45^ In this study, foretinib exhibited a marked survival benefit in the c‐Met overexpressing peritoneal dissemination mouse xenograft model that has striking similarities with clinical characteristics and progression pattern of GAC. Additionally, foretinib exhibited a strong antitumour response in different subcutaneous GAC models, and as expected, foretinib had higher antitumour activity in c‐Met overexpressing MKN‐45 xenografts compared with c‐Met low‐expressing SNU‐1 xenografts. The divergent levels of c‐Met expression in different GAC cells have previously been reported.^46^ Antitumour activity of foretinib in SNU‐1 xenografts can be explained by its inhibitory effect on other oncogenic pathways including VEGFR2, PDGFR‐β, Axl, c‐Kit, Flt‐3 and Tie2 in tumour epithelial cells or a possible c‐Met activity in non‐epithelial tumour stromal cells. Previous clinical studies using c‐Met inhibitors that only showed modest antitumour activity in metastatic GAC patients may likely be due to the agent's activity being more restricted to the c‐Met signalling pathway.^23^, ^24^ In addition, a clinical study reported that c‐Met and VEGFR2 signalling are major drivers in the progression of some GACs, indicating the therapeutic potential of concurrently blocking these two oncogenic pathways.^47^ The tumorigenic activities of all the potential targets of foretinib including c‐Met, VEGFR2, PDGFR‐β and Axl are well‐established. The antitumour benefits of foretinib as a single‐agent and combined with nanoparticle paclitaxel in different preclinical models indicate its direct impact on other oncogenic pathways apart from the c‐Met pathway. Given the fact that c‐Met overexpression has a crucial role in single‐agent VEGFR inhibitor resistance,^32^ simultaneous blockage of these two pathways may also have benefits in avoiding or delaying resistance development. Thus, the results of this study highlight the potential of dual targeting of c‐Met and VEGFR2 in GAC therapy.

*Nab*‐paclitaxel exhibited greater antitumour activity as opposed to other common chemotherapies in GAC, such as oxaliplatin in this study.^9^, ^39^, ^48^ Some benefits of *nab*‐paclitaxel can be accredited to its enhanced permeability and retention, better penetration and distribution in the tumour directing its amplified anti‐mitotic effects in cancer epithelial and stromal cells.^49^, ^50^ Based on its anti‐stromal activity, *nab*‐paclitaxel has previously been shown to be a compelling agent in the combination therapy approach possibly by enhancing tumour penetration, bioavailability and retention of other drugs in combination.^39^, ^48^, ^51^ It is plausible that distinct mechanisms of action of foretinib and *nab*‐paclitaxel determine their ability to produce combination benefits. *Nab*‐paclitaxel's well‐known anti‐stromal and anti‐mitotic responses can primarily be associated with its impact on cancer cell proliferation reduction, apoptosis induction and depletion of stromal density,^52^, ^53^ whereas foretinib not only decreased tumour cell proliferation or increased apoptosis but it also markedly depleted tumour vasculature. Although the definitive mechanisms of additive benefits of foretinib plus *nab*‐paclitaxel therapy remain elusive, some influential factors might include tumour vasculature normalization, enhanced drug delivery into the tumour, depletion of stroma density and probably direct addition to the cytotoxic response by foretinib blocking tumorigenic processes such as proliferation, invasion, metastasis and EMT.^51^, ^54^

In vitro cell viability analysis of mutationally distinct GAC cells indicated that foretinib caused a smaller anti‐proliferative effect on the c‐Met low‐expressing SNU‐1 cell line compared with c‐Met overexpressing cell lines MKN‐45 or KATO‐III. Immunoblot analysis revealed that the foretinib antitumour effect corresponded with its specific and predicted anti‐proliferative, anti‐angiogenic and pro‐apoptotic activity in upstream receptor protein c‐Met and downstream PI3K/MAPK and Bcl‐2 signalling proteins.

Aggressive GAC growth is dependent on multiple factors including tumour cell proliferation, adhesion, invasion, angiogenesis and migration. Consequently, a compelling therapeutic drug combination should be able to affect most of these tumorigenic mechanisms with favourable toxicity. Based on the fact that c‐Met and VEGFR2 pathways are involved in most of the GAC growth and progression factors, dual inhibition of these two pathways by new‐generation multitarget RTK inhibitors such as foretinib with specific targeting profiles and favourable toxicity have imperative potential to advance GAC therapeutic approaches. This study demonstrates that the dual inhibition of c‐Met and VEGFR2 pathway is an effective therapeutic strategy for GAC and it has the ability to augment the response of a new‐generation chemotherapy *nab*‐paclitaxel, thereby providing additional avenues to ameliorate clinical GAC therapy.

## CONFLICT OF INTEREST

The authors declare no conflict of interest.

## AUTHOR CONTRIBUTIONS

**Meghan Grojean:** Data curation (lead); Formal analysis (supporting); Funding acquisition (supporting); Methodology (supporting). **Margaret A Schwarz:** Data curation (supporting); Formal analysis (supporting). **Johann Schwarz:** Data curation (supporting); Formal analysis (supporting). **Md Sazzad Hassan:** Data curation (supporting); Formal analysis (supporting). **Urs von Holzen:** Writing‐review & editing (supporting). **Changhua Zhang:** Data curation (supporting); Formal analysis (supporting). **Roderich E Schwarz:** Funding acquisition (supporting); Writing‐review & editing (supporting). **Niranjan Awasthi:** Conceptualization (lead); Data curation (supporting); Formal analysis (supporting); Funding acquisition (supporting); Investigation (supporting); Methodology (supporting); Supervision (lead); Writing‐original draft (lead).

## Supporting information

Fig S1

Fig S2

Table S1
