# Supplementary figures and images for: Targeted dual inhibition of c‐Met/VEGFR2 signalling by foretinib improves antitumour effects of nanoparticle paclitaxel in gastric cancer models
Source: J Cell Mol Med. 2021 May 3;25(11):4950–61. doi: 10.1111/jcmm.16362 (PMC8178268; doi:10.1111/jcmm.16362)

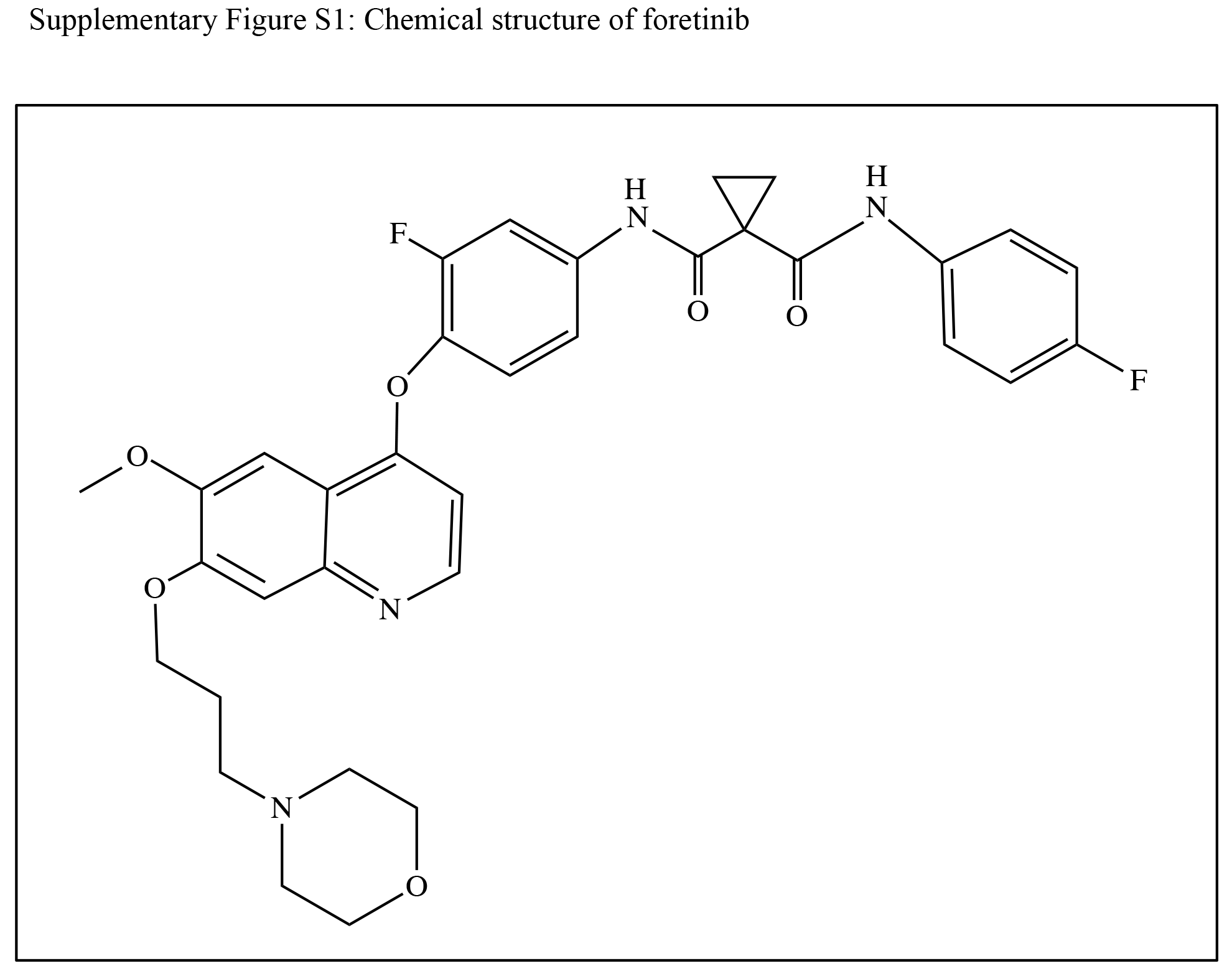

Supplement: Supplementary file 1 — Fig S1 [file JCMM-25-4950-s003.tif]

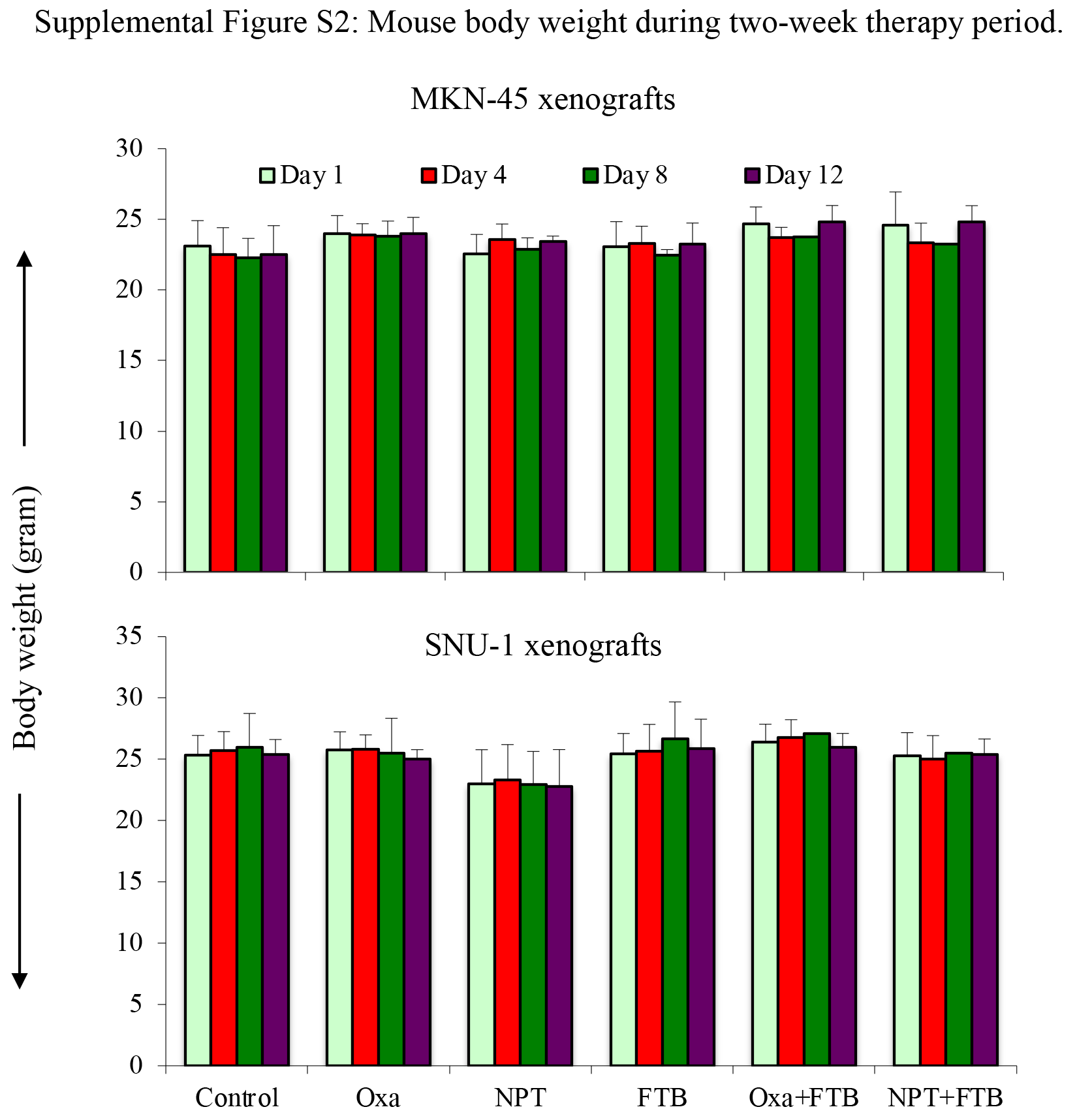

Supplement: Supplementary file 2 — Fig S2 [file JCMM-25-4950-s002.tif]

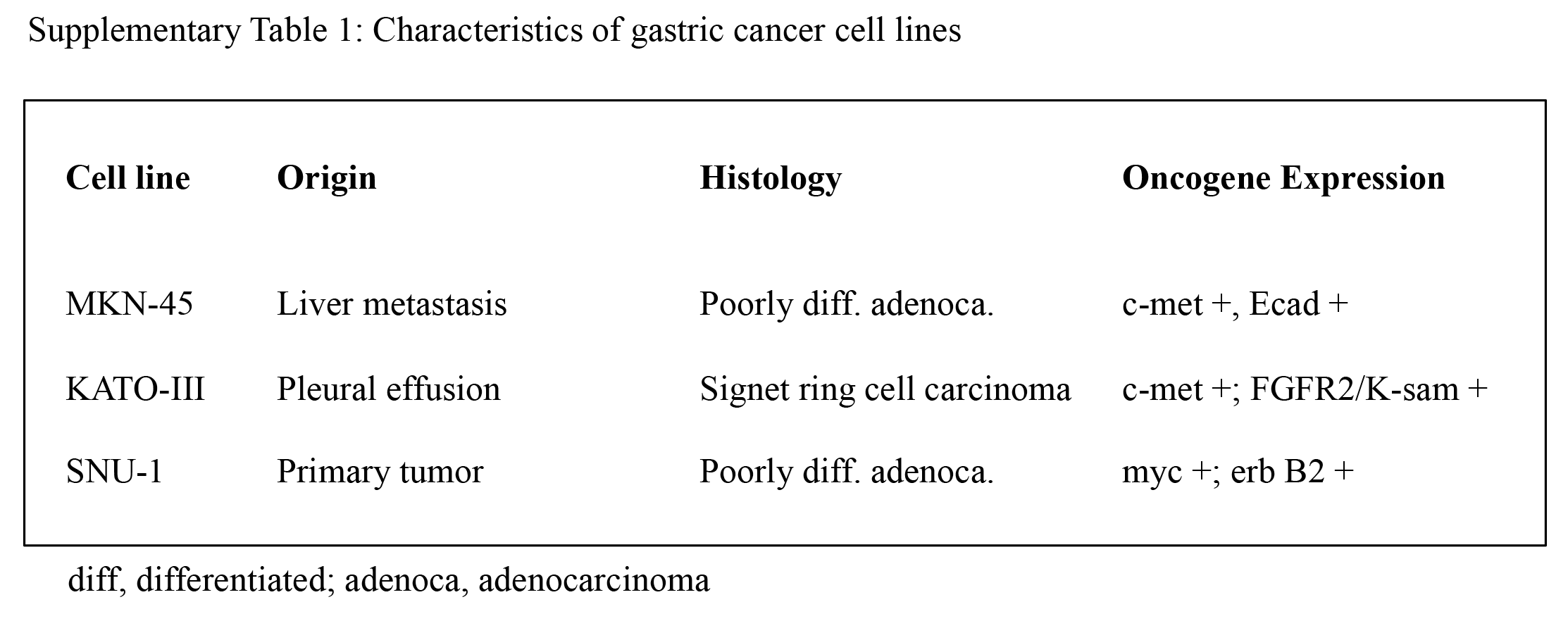

Supplement: Supplementary file 3 — Table S1 [file JCMM-25-4950-s001.tif]
